# Use of rituximab in mature, high-grade and advanced-stage pediatric B-lineage non-Hodgkin lymphomas: a systematic review, meta-analysis and the Brazilian reality

**DOI:** 10.3389/fped.2025.1532274

**Published:** 2025-01-20

**Authors:** Alejandra Adriana Cardoso de Castro, Liana Alves de Oliveira, Diancarlos Pereira de Andrade, Edna Kakitani Carbone, Roberto Rosati

**Affiliations:** ^1^ Department of Pediatric Oncology and Hematology, Pequeno Principe Hospital, Curitiba, Brazil; ^2^ Faculdades Pequeno Príncipe, Curitiba, Brazil; ^3^ National Science and Technology Institute for Children’s Cancer Biology and Pediatric Oncology, INCT BioOncoPed, Porto Alegre, Brazil; ^4^ Pelé Pequeno Príncipe Research Institute, Curitiba, Brazil

**Keywords:** rituximab, lymphoma, non-Hodgkin lymphoma, B cell lymphoma, pediatrics

## Abstract

**Objectives:**

Rituximab is a valuable agent for treating adult B-cell non-Hodgkin lymphoma (B-NHL), and several studies have tested its efficacy in children with mature, high-grade B-NHL. The aim of the present study was to systematically review the use of rituximab in children and adolescents with high-grade mature B-NHL and to conduct a meta-analysis of the evidence. Since access to this medication in public health systems in low- and middle-income countries is complex, we were also interested in mapping access to it in Brazil.

**Methods:**

We conducted a systematic review and meta-analysis on the survival of pediatric patients with mature, high-grade and advanced-stage B-NHL treated with rituximab in combination with chemotherapy in first-line treatment or later. Patients' access to the medication was evaluated through a questionnaire sent to oncologists in Brazilian pediatric oncology centers.

**Results:**

We selected 17 trials, which were subsequently grouped by disease type and line of therapy. In patients receiving first-line treatment, excluding those with primary mediastinal B-cell lymphoma (PMBL), the use of rituximab resulted in (1) better event-free survival [Hazard Ratio of 0.37 (0.22, 0.61); *p* < 0.01]; (2) a reduced risk of events [odds ratio of 0.44 (0.26–0.76); *p* = 0.003]; and (3) a reduced risk of death [odds ratio of 0.44 (0.21–0.89); *p* = 0.02]. In refractory or relapsed (R/R) patients, rituximab use was associated with a decreased chance of death [odds ratio of 0.25 (0.09–0.75); *p* = 0.01]. Additionally, our survey included 31 Brazilian centers, 63% of which reported bearing the cost of rituximab.

**Conclusion:**

Rituximab improves outcomes in pediatric patients receiving first-line treatment for high-grade B-NHL (except PBML) and overall survival in R/R patients. However, access to rituximab in Brazilian hospitals is currently dependent on centers supporting its economic burden.

**Systematic Review Registration:**

https://www.crd.york.ac.uk/prospero/, PROSPERO (CRD42021292912).

## 1 Introduction

Lymphomas, the third most common cancer in pediatric patients (1, 2), are commonly divided into two categories: Hodgkin lymphoma (HL) and non-Hodgkin lymphoma (NHL). NHL includes a group of mature B lymphomas, comprising Burkitt Lymphomas (BL) and Diffuse Large B-Cell Lymphomas (DLBCL), responsible for approximately 60% of the total lymphomas in children and adolescents (2–5), with a greater prevalence in males (3). Furthermore, BL and DLBCL are characterized by strong CD20 expression (4, 6–9), and despite having aggressive behavior (6), the response to intensive short-term chemotherapy is very good, reaching a cure rate of more than 85% (1, 2, 9–12), even when patients are diagnosed in advanced stages of the disease.

This group also includes Primary Mediastinal B-cell Lymphomas (2%), which have similar aggressiveness but lower survival rates and higher frequencies in female patients (13, 14), and indolent lymphomas, which are rare in children and adolescents.

Despite the progressive advances in first-line protocols, patients with relapsed or refractory disease have an expressive reduction in overall survival (OS) (1, 4, 5, 9–11, 15), which is usually below 30% (7).

Rituximab is a chimeric monoclonal antibody that targets the CD20 lymphocyte surface antigen, which is expressed in more than 98% of high-grade mature B lymphomas (16), causing apoptosis and cytotoxicity (7, 14, 17). In clinical trials set in place thus far, rituximab in the pediatric population has been added to conventional chemotherapy and is reserved for high-grade mature B lymphomas and advanced-stage or high-risk populations, in addition to refractory/relapsed patients. Some studies already demonstrate its efficacy and safety, like those described in Cairo and Beishuizen's (18) review and Goldman et al. (19) trial. In this latter study (19), no serious adverse events were classified as probably or definitely caused by rituximab, and there was an increase in event-free survival (EFS) compared with a historic control not treated with rituximab (95% vs. 84%). Cairo (7) reported a 2-year EFS above 95% for intermediate-risk patients when rituximab was associated with chemotherapy treatment and a 2-year EFS of 90% for high-risk patients compared with the historic control. Moreover, the study of Minard-Colin et al. (20) prematurely ended because of the advantage of 13 percentage points in 1-year EFS for patients who received rituximab in contrast to treatment with chemotherapy alone.

Unfortunately, low-income countries have restrictions regarding the use of rituximab, the main reason being its substantial price. In Brazil, most of the population receives treatment through the public health system, and the allocated amount for the treatment of children with lymphomas is insufficient to add rituximab to conventional chemotherapy.

Therefore, the present study aims to evaluate the evidence that supports the use of rituximab in children/adolescents with high-grade mature B-NHL and map its application among Brazilian cancer treatment centers.

## 2 Methods

For this systematic review, our guiding question was “Does the use of rituximab in addition to standard chemotherapy improve overall and event-free survival rates in pediatric patients affected by high-grade, mature and advanced-stage B-cell non-Hodgkin lymphoma as first-line treatment or in the treatment of relapsed/refractory disease?”. Following the PICO strategy, searches were made in literature databases with selected terms joined by Boolean operators, with refinements when allowed by the database. The detailed information is available in the Supplementary Materials.

The inclusion criteria were as follows: studies with patients between 0 and 21 years of age who had mature and aggressive non-Hodgkin lymphomas of the B lineage, with CD20 expression, including BL or Burkitt-like, DLBCL or B-NHL, not otherwise specified (NOS) in advanced stages (III and IV) or refractory/relapsed, or primary mediastinal B lymphoma (PMBL) in any stage, that used rituximab combined with conventional chemotherapy, ideally (but not essentially) compared with conventional isolated chemotherapy, allowing additional treatment, such as bone marrow transplant, in both groups.

The exclusion criteria were as follows: trials that did not evaluate survival with the treatment used (with or without rituximab); trials that included patients with indolent lymphomas or immunocompromised patients; trials that used intrathecal rituximab or less than four doses applied parenterally (except, in this case, patients in second-line or later treatment); trials that included patients who had primary central nervous system lymphomas; studies without adequately described methodology; “case report” and “review” articles; studies in which biases that could compromise data extraction were detected; or studies that were available in their complete form in languages that were not English, Portuguese, Spanish or French.

Owing to the low availability of studies on this topic in the pediatric population, the present review included retro- and prospective studies, which were randomized or not randomized. Single-arm trials were included only when rituximab was used.

The project was submitted and approved by the PROSPERO platform (register number CRD42021292912).

All search, evaluation, selection, data extraction and risk of bias evaluation processes were independently conducted by two researchers. Discrepancies were evaluated by a third researcher.

Statistical analysis and forest plot creation were performed via R versions 4.0.2 and 4.3.0 (21). The meta-analysis was conducted with the “meta” package version 7.0-0 (22); odds ratio calculations with 95% confidence intervals (CI) were performed via a fixed effect model, and hazard ratio calculations with 95% CI were performed via common and random effects models.

The risk of bias was determined via the Cochrane network's, Risk Of Bias In Non-randomized Studies of Interventions (ROBINS-I) (23) and Risk of Bias 2 (RoB 2.0) (24) tools. RoB 2.0 was used in randomized trials. In domain 2 of the evaluation, we considered the effect of attribution to the intervention. To evaluate non-randomized single-arm trials, the ROBINS-I tool was adapted without considering the third criterion (“bias in the classification of interventions”), with the remaining criteria normally examined.

Furthermore, an online questionnaire on the use of rituximab was offered to pediatric oncologists affiliated with the Brazilian Society of Pediatric Oncology (SOBOPE). The purpose of this research was to evaluate patients' access to the medication in question. The questions referred to current practices in the treatment center where the pediatrician was practicing, and all the data were analyzed on a treatment center basis. This research was approved by the IRB board of our institution (CAAE 48264421.3.0000.5580).

## 3 Results

**Figure 1:**
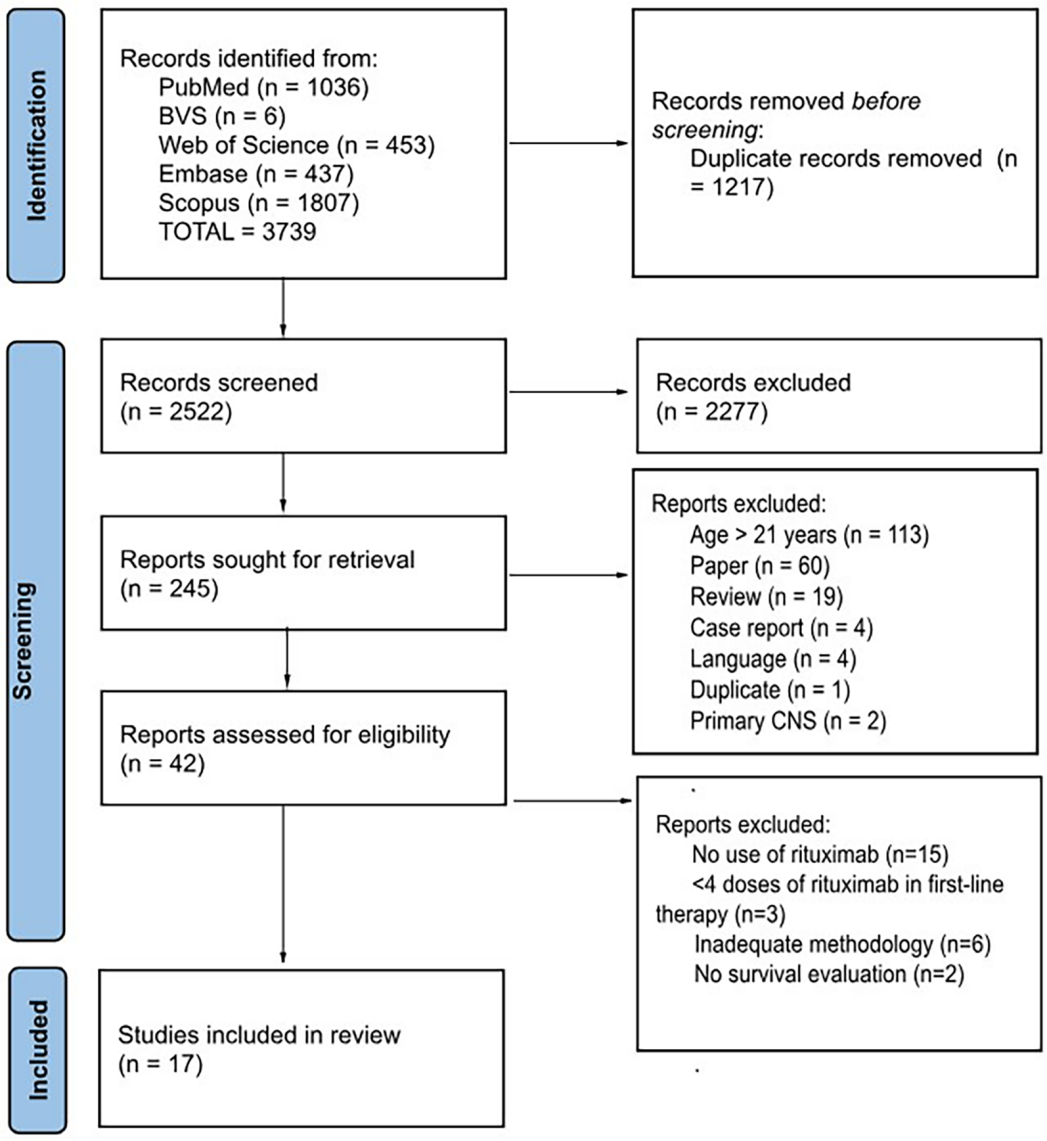
The search and selection of articles were carried out according to the PRISMA (25) standard. One of the articles included in the systematic review [Jourdain et al. (26)] did not follow this flow because it was not selected during the search of the databases but rather was identified during the literature review and subsequently included.

The 17 selected trials (Figure 1) were separated into three groups for adequate evaluation: patients receiving first-line therapy with BL, Burkitt-like, DLBCL and mature B-NHL, NOS; patients receiving first-line treatment with PMBL; and patients with refractory or relapsed disease receiving second-line or later treatment.

### 3.1 Patients in first-line treatment with BL, burkitt-like, DLBCL and B-NHL, NOS

Seven trials, detailed in Table 1, published between 2014 and 2021, were included in this group, with a total of 775 patients aged between 0 and 17 years. Among these 775 patients, 631 were male and 144 were female, 264 had stage III disease, and 511 had stage IV disease. In Samochatova et al. (27) and Minard-Colin et al. (12), seven of these patients had other diagnoses after review but could not be withdrawn from the analysis (1 B-acute lymphoblastic leukemia, 6 PMBL). Among these trials, three described performing a central review of diagnoses (12, 27, 32), and it is important to highlight that Minard-Colin et al. (12) accounted for more than 42% of the total sample included in this evaluation group, and was the only randomized phase III trial [while Samochatova et al. (27) and Fu et al. (28) evaluated the intervention arm prospectively, they were compared with historical controls].

**Table 1:** General data, event-free survival (EFS) and overall survival (OS) in selected trials of patients undergoing first-line treatment for mature, high-grade and advanced-stage B-non-Hodgkin lymphoma, with the exception of primary mediastinal B-cell lymphoma (PMBL).

	Samochatova et al. (27)	Fu et al. (28)	Mussolin et al. (29)	Minard-Colin et al. (12)	Zhen et al. (30)	Zhu et al. (31)	Huang et al. (32)
Characteristics of the trial	Prospective, non-randomized, without number of centers	Prospective, non-randomized, unicentric	Retrospective, non-randomized, without number of centers	Prospective, randomized phase III trial, multicentric	Retrospective, non-randomized, unicentric	Retrospective, non-randomized, unicentric	Retrospective, non-randomized, unicentric
Sample with/without rituximab (*n*)	83/0	46/23	21/81	164/164	41/49	13/12	69/9
Sex M/F (%)	75.9/24.1	76.8/23.2	83.3/16.7	82.9/17.1	82.2/17.8	76/24	83.3/16.7
Age (median/mean)	8.84/ND	ND/6.9	8/ND	ND/8.9	7/ND	8/ND	5.7/ND
Follow-up (months)	44.8–77.2	1–88.8	4.8–165.6	ND	1–96	2.5–158	1–72
Sample stages III/IV (*n*)	38/45	53/16	0/102	144/184	29/61	0/25	0/78
Evaluated time (years)	3	5	4	3	3	5	3
Events in the group with rituximab (*n*)	ND	6	5	10	2	ND	ND
Events in the group without rituximab (*n*)	NA	4	19	28	8	ND	ND
EFS with rituximab (%)	84	83.7	76	93.9	96.8	ND	76.1
EFS without rituximab (%)	NA	69.6	76	82.3	82.3	83.3	33.3
*p* value (EFS)	NA	0.1062	ND	0.00096	0.077	ND	0.002
Deaths in the group with rituximab (*n*)	10	6	ND	8	ND	ND	ND
Deaths in the group without rituximab (*n*)	NA	4	ND	20	ND	ND	ND
OS with rituximab (%)	82	ND	ND	95.1	96.7	84.6	ND
OS without rituximab (%)	NA	ND	ND	87.3	85.1	82.5	ND
*p* value (OS)	NA	NA	NA	ND	0.129	0.91	NA

*n*, occurrence number; M, male; F, female; ND, not described; NA, not applicable.

The chemotherapy protocols used, described as originally mentioned, were: AIEOP LNH-97 (Mussolin et al.) (29), modified B-NHL-BFM 95 (Zhen et al.) (30), B-NHL-BFM 90/95 [Fu et al. (28); Zhu et al. (31)], modified B-NHL-BFM 90 (Samochatova et al.) (27), BCH B-cell non-Hodgkin's lymphoma regimen (Huang et al.) (32) and modified FAB/LMB 96 (Minard-Colin et al.) (12).

Five of the 7 protocols used 6 doses of rituximab as the maximum number [exceptions: Samochatova et al. (27) and Fu et al. (28), both with 4 doses], and the number of patients who received this monoclonal antibody was 437, whereas 338 did not. Only one trial (Fu et al.) (28) used radiotherapy for some patients as associated treatment.

**Figure 2:**
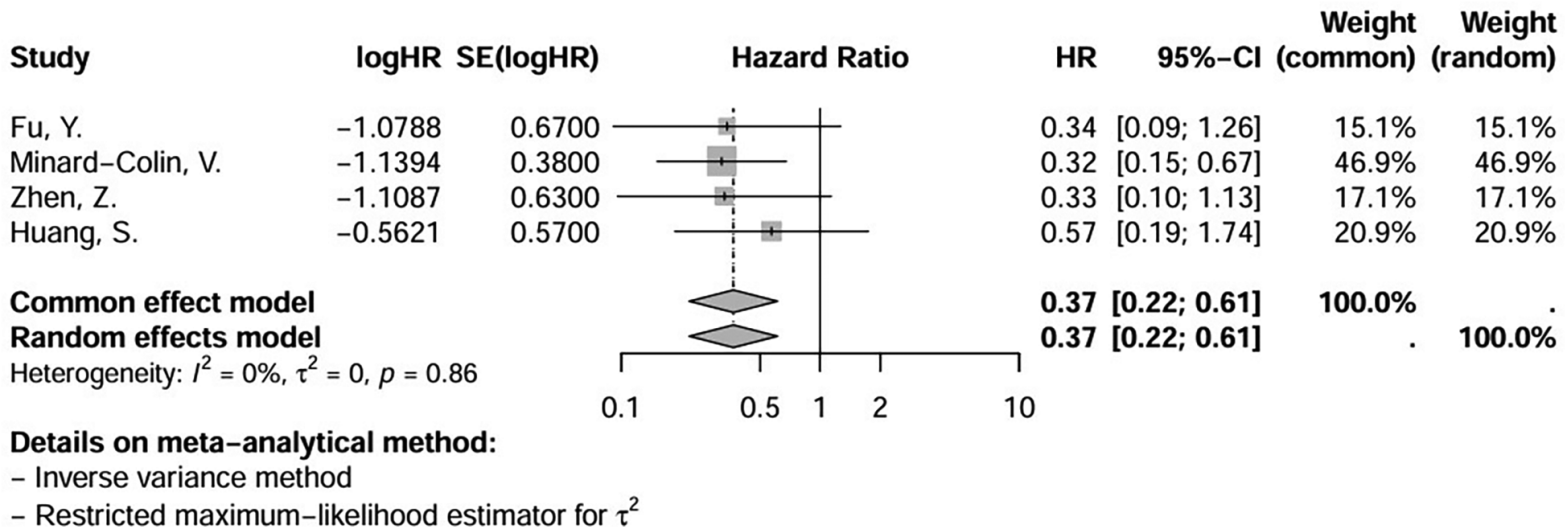
Hazard ratio (HR) of event-free survival (EFS) in patients receiving first-line treatment for mature, high-grade and advanced-stage B-non-Hodgkin lymphoma (NHL), except for primary mediastinal B-cell lymphoma (PMBL), showing the weight of each trial and a forest plot. Significant overall *p*-value (<0,01). (logHR) = ln(log e).

**Figure 3:**
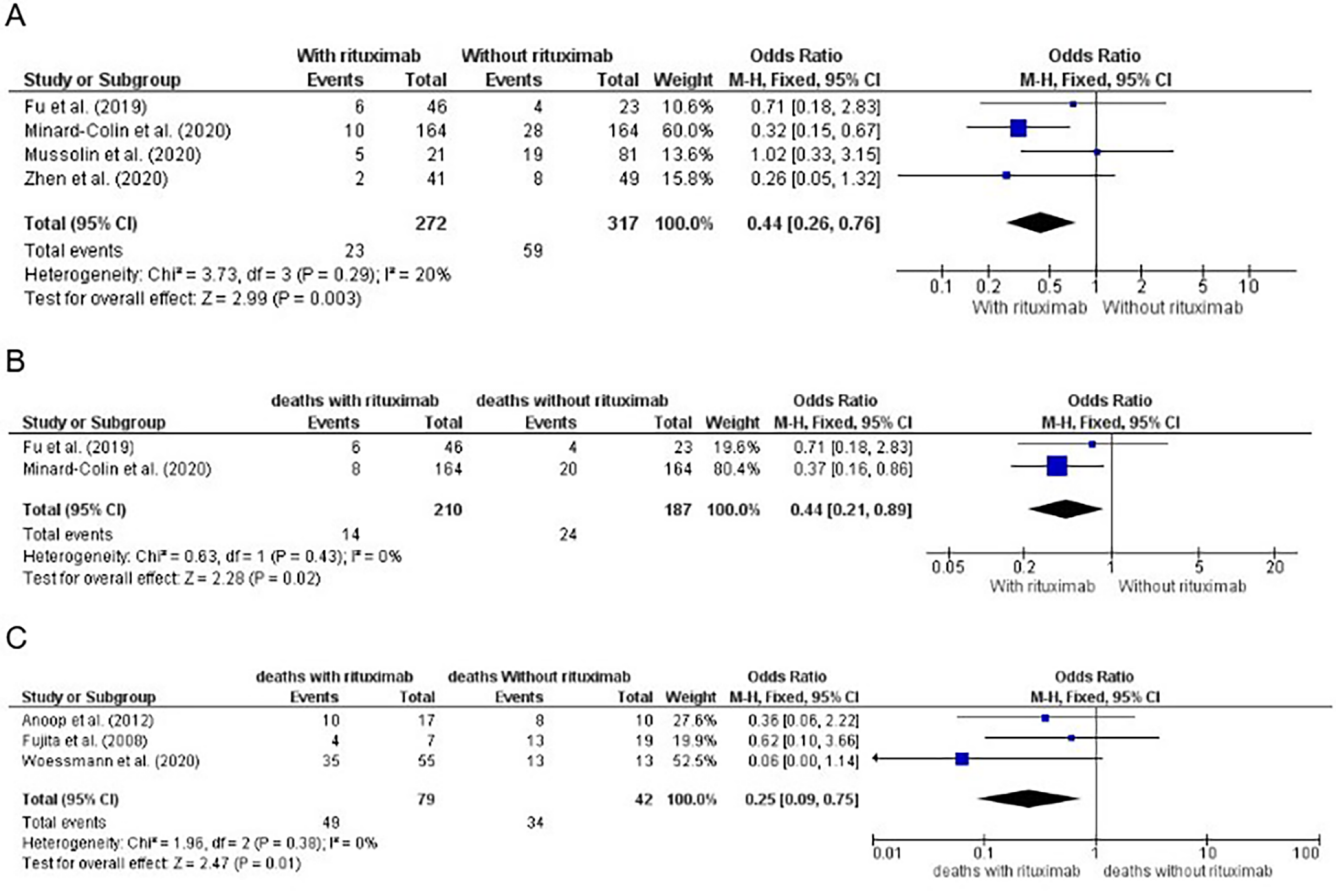
**(A)** Number of events by treatment arm of patients in first-line treatment for mature, high-grade and advanced-stage B-non-Hodgkin lymphoma (NHL), except primary mediastinal B-cell lymphoma (PMBL), for calculation of the odds ratio (OR), showing the weight of each trial, and forest plot. Significative *p* value (0.003). **(B)** Number of deaths by treatment arm of patients in first-line treatment for mature, high-grade and advanced-stage B-NHL, except PMBL, for calculation of the OR, showing the weight of each trial, and forest plot. The significant *p* value was 0.02. **(C)** Number of deaths by treatment arm of patients with relapsed/refractory disease for calculation of the OR, showing the weight of each trial, and a forest plot. Significant *p* value (0.01).

We included in the meta-analysis all trials with two treatment arms in which the number of patients who used or did not use rituximab and their respective events and deaths were provided or were calculable through information provided. The meta-analysis results for this group are shown in Figures 2, 3A, B. With respect to hazard ratio (HR) analysis, our data indicate that the selected studies are strongly consistent (*I*^2^ of 0% and tau^2^ of 0); differences between the study results were not statistically significant (*p* = 0.86). The combined HR of 0.37 with a 95% CI of (0.22, 0.61) and a *p*-value < 0.01 shows that there is a statistically significant reduction in risk (63%) for the event of interest in the treatment group compared with the control group. Overall, the meta-analysis suggested a strong and consistent treatment effect across the studies, with no significant heterogeneity.

### 3.2 Patients in first-line treatment with PMBL

Four articles published between 2017 and 2022 were selected for this subgroup, with patients aged between 7 and 21 years. The total number of patients was 138, with 62 males and 76 females. Notably, some trials staged patients according to the Ann Arbor system and others according to St. Jude's/Murphy classification (Table 2). For the previous group, during central pathology review, 2 patients with changes in diagnosis were identified (one with DLBCL and the other with gray-zone lymphoma). These 2 patients could not be excluded from the analysis.

**Table 2:** General data, event-free survival (EFS) and overall survival (OS) in selected trials of patients undergoing first-line treatment for primary mediastinal B-cell lymphoma (PMBL).

	Giulino- Roth et al. (33)	Pillon et al. (34)	Burke et al. (35)^a^	Dourthe et al. (36)
Characteristics of the trial	Retrospective, multicentric	Retrospective, multicentric	Prospective, multicentric	Prospective, multicentric
Sample with/without rituximab (*n*)	38/0	12/0	46/0	21/21
Sex M/F (%)	44.7/55.3	58.3/41.7	43/57	43/57
Age (median)	16	16	15.4	15
Follow-up (months)	2.7–101	15.6–109.2	52.6–69.2	ND
Sample stages (*n*) I/II/III/IV	1/9/23/5	0/0/12/0	0/31/1/12	0/19/1/22
Staging system	Ann Arbor	SJ/Murphy	Ann Arbor	Ann Arbor
Evaluated time (years)	3	4.8	4	5
Events in the group with rituximab (*n*)	ND	2	14	1
Events in the group without rituximab (*n*)	NA	NA	NA	4
EFS with rituximab (%)	81	83.9	69.6	95.2
EFS without rituximab (%)	NA	NA	NA	81
*p* value (EFS)	NA	NA	NA	ND
Deaths in the group with rituximab (*n*)	ND	1	7	0
Deaths in the group without rituximab (*n*)	NA	NA	NA	2
OS with rituximab (%)	90.7	91.7	84.8	100
OS without rituximab (%)	NA	NA	NA	90.5
*p* value (OS)	NA	NA	NA	ND

*n*, occurrence number; M, male; F, female; ND, not described; NA, not applicable.

^a^ Two patients had their diagnosis changed after pathological review.

Two of these trials included pathological review of diagnoses [Burke et al. (35) and Dourthe et al. (36)], and all are non-randomized.

The chemotherapy protocols that were used were modified AEIOP-NHL-97 (Pillon et al.) (34), DA-EPOCH-R [Giulino-Roth et al. (33); Burke et al. (35)] and LMB 2001 [Dourthe et al. (36)], with six to eight doses of rituximab when used (*N* = 117). One trial [Giulino-Roth et al. (33)] employed radiotherapy for a group of patients.

Details of the extracted data for this group can be found in Table 2. Meta-analysis was not possible since only one of the selected sources had two treatment arms.

### 3.3 Patients with refractory and relapsed disease

In this group, 6 articles published between 2008 and 2020 (Table 3) with patients between 1 and 20 years of age were selected. A total of 241 patients were included, with 91 male and 29 female patients described [3 trials without this differentiation reported: Fujita et al. (37); Anoop et al. (38); Woessmann et al. (39)]. The types of lymphoma included in this analysis were Burkitt lymphoma/leukemia, DLBCL, PMBL and mature high-grade B-NHL, NOS.

**Table 3:** General data, event-free survival (EFS) and overall survival (OS) in selected trials of patients who underwent second-line treatment (or later) for mature and high-grade B-non-Hodgkin lymphoma.

	Fujita et al. (37)^a^	Griffin et al. (9)	Anoop et al. (38)	Jourdain et al. (26)	Osumi et al. (15)	Woessmann et al. (39)
Characteristics of the trial	Retrospective, multicentric	Prospective, multicentric	Retrospective, multicentric	Retrospective, multicentric	Retrospective, multicentric	Retrospective, multicentric
Sample with/without rituximab (*n*)	7/19	20/0	17/10	16/51	28/5	55/13
Sex M/F (%)	ND	80/20	ND	75/25	75.8/24.2	81/19
Age (median)	13	ND	9.4	9.6	9.7	9.6
Follow-up (months)	ND	1–30	14.4–76.8	Max. 204	ND	19.2–115.2
Was there use of rituximab in first-line?	ND	ND	No	No	No	Some patients
Evaluated time (years)	4	3	10	5	5	4
Events in the group with rituximab (*n*)	ND	13	ND	ND	ND	35
Events in the group without rituximab (*n*)	ND	NA	ND	ND	ND	13
EFS with rituximab (%)	ND	ND	ND	ND	ND	ND
EFS without rituximab (%)	ND	NA	ND	ND	ND	ND
*p* value (EFS)	NA	NA	NA	NA	NA	NA
Deaths in the group with rituximab (*n*)	4	12	10	ND	16	35
Deaths in the group without rituximab (*n*)	13	NA	8	ND	NAD	13
OS with rituximab (%)	ND	37.5	ND	43.8	ND	36
OS without rituximab (%)	ND	NA	ND	25.5	ND	0
*p* value (OS)	ND	ND	0.11 (min. 2 doses of rituximab)/0.006 (min. 4 doses)	0.29 (uni) 0.1 (multi)	ND	0.016

*n*, occurrence number; M, male; F, female; Max, maximum; min., minimum; ND, not described; NA, not applied; NAD, not adequately described.

^a^ Three patients without data about the treatment that was used were excluded from the analysis, 3 patients with no description of pretransplant chemotherapy or radiotherapy and one patient treated with palliative intent.

Only one of these trials [Griffin et al. (9)] used a single chemotherapy protocol for rescue treatment (ICE—ifosfamide, carboplatin and etoposide). Randomization was not performed in any of them, whereas central review was performed in Fujita et al. (37), Anoop et al. (38), Jourdain et al. (26) and Woessmann et al. (39). Importantly, in Jourdain et al. (26), two of the 67 patients did not receive standard treatment because one of them underwent only complete surgical resection of the neoplasm, and the other patient received palliative care but could not be excluded from the analysis.

The number of rituximab doses used ranged between 1 and 6 since the intention in this group of patients is to take them to consolidation treatment as soon as they reach remission. In this regard, all trials included autologous bone marrow transplant (BMT) as a therapeutic option and, consequently, the previous use of conditioning chemotherapy, with the use of allogenic BMT for some patients in 5 of the selected trials [exception: Anoop et al. (38)] and radiotherapy in three trials [Fujita et al. (37); Jourdain et al. (26); Osumi et al. (15)].

The meta-analysis results for this group, considering the number of deaths, are shown in Figure 3C.

### 3.4 Bias analysis

The analysis of both outcomes (overall survival and event-free survival) was performed separately; however, their results coincided when applicable and are described on a unified basis (Supplementary Tables S1, S2).

### 3.5 Survey with pediatric oncologists in Brazil

The survey was performed with the intention of understanding the current Brazilian reality regarding the availability and use of rituximab for pediatric (0–21 years) patients with high-risk B-NHL and patients with refractory or relapsed disease. The following results were obtained: regarding the locations of the participating hospitals in Brazil, the majority were in the southern (35.5%, 11 in total) and southeastern (9 responses, 29%) regions, followed by the north (16,1%, 5), northeast (12,9%, 4) and finally the midwest (6,5%, 2 collaborations) regions. Interestingly, 93.6% of the centers (29/31) reported treating patients through the Brazilian public health system. Of those, 48.4% exclusively treat these patients, while 45.2% also treat insured patients. Two of the centers treat exclusively insured clients.

The average number of new cases of high-grade mature B-NHL treated per year in each center was as follows: less than 5 in almost half of the sample (48.4%, 15 institutions), between 5 and 10 in 41.9% (13) and between 10 and 20 in only 6.5% (2).

Protocols used in patients undergoing first-line treatment (with or without rituximab and not taking into account patients with PMBL) included LNH 2016 (Brazilian protocol) in 15 institutions, FAB/LMB 96 (or similar) in 7, NHL-BFM-95 (or similar) in 6, SBJC3 (or similar) in 2 and CHOP in one hospital.

In high-risk patients undergoing first-line treatment, one of the centers reported not using rituximab, whereas two reported using it only for insured patients. Two centers reported the need for legal action against the government to obtain the drug. The remaining centers (83.9%) reported the use of rituximab in all patients. When asked about the motive for not using rituximab, or for using it only for patients who are not treated by the public health system, the answer was unanimous: the high cost of the medication. This acquisition difficulty was also mentioned in Costa et al. (40), in which 30% of 97 Brazilian centers reported the unavailability of rituximab for patients with high-risk B-NHL undergoing first-line treatment. Strasser-Weippl et al. (41) also cited, in their report for The Lancet Oncology Commission, the growing request for high-cost medications through the courts in Latin American countries, including Brazil, generating supply failures, even if temporary.

The most commonly used second-line treatments (with or without rituximab) were ICE (ifosfamide, carboplatin and etoposide) in 18 institutions and NHL-BFM-95 reinduction (CC/AA) in 2 institutions, with no answers from 3 of the participants. In relapsed/refractory patients, drug use is even lower: 21 centers (67.7%) use rituximab for all patients in this situation, three obtain it judicially (9.7%), the other three do not employ it, and two did not answer (6.5%); one uses it only for patients with health insurance, and one service reported using it occasionally, without further descriptions (3.2%). With respect to the reason for the selective administration of rituximab (or the total lack of use), three participants reported a high cost, and the other was motivated by the limited scientific evidence in the pediatric population.

Finally, considering that the cost of rituximab surpasses the monthly budget that the public health system offers for these patients' treatment, the last question asked who finances the use of rituximab in the addressed situation: 63.3% of the participating institutions reported that they pay for rituximab (19/31). Among the remaining institutions, 16.7% (5 services) reported that their patients file lawsuits against the public health system so that financing can be obtained via public resources—two of the 5 institutions that provided this answer to the question reported using rituximab for all patients in first-line treatment. In the second-line, one department stated that the medication is used for all; the other stated that it is only used for patients with health insurance. Additionally, 13.3% (4 institutions) rely on the collaboration of non-governmental organizations, and 6.7% (2 institutions) employ governmental funding.

## 4 Discussion

Our review and meta-analysis showed evidence of the benefits of using rituximab for treating children and adolescents with advanced-stage or relapsed/refractory high-grade mature B-NHL, consistent with the outcome of the only phase III study available for this purpose until now, in advanced-stage patients on first-line treatment [Minard-Colin et al. (12), included in this meta-analysis]. There is a great difficulty in conducting randomized prospective trials in patients with pediatric lymphomas, owing to the lower prevalence than in adult patients. Furthermore, there were several difficulties in paper selection, starting with the lack of standardization regarding the age of this population: there are trials that include, in pediatric studies, patients between 15 and 39 years, the AYA population. In this last circumstance, some of the articles that were found had to be excluded from the present review due to the impossibility of separating the data of the population that is our interest (patients up to 21 years old).

Another difficulty, which is a potential cause of bias, is the use of many different protocols for the treatment of the same pathology, several of which suffer from modifications from its original form that are not always described for the reader's assessment. Furthermore, the lack of stratification regarding the risk of patients evaluated in most trials caused us to alter our inclusion criteria for patients with advanced-stage disease, instead of those with high-risk disease, as was the original intention.

Additionally, some trials compared the use of rituximab with a historical control from the same research group that did not include this agent. Here, a potential bias can be identified, since protocols undergo modifications over time, and overall support for these patients tends to improve in this interval, which privileges the arm treated more recently.

The present review also aimed to evaluate whether the inclusion of rituximab in the treatment protocol increased the risk of serious infections or toxic death, but that was not possible because of the lack of these data in most of the included trials. Fu et al. (28) described early death in each group, or 2.2% in the group that used rituximab, in contrast with 4.3% in the group without rituximab. Minard-Colin et al. (12) reported a greater number of infections of Grade 4 or higher in the group treated with rituximab (18.5% vs. 11.1%), with a *p*-value of 0.07, but the rate of toxic deaths was 1.8%, with an equal distribution among both groups. Dourthe et al. (36) did not report any death by toxicity in either group in the trial. In the remaining double-armed clinical trials, when there was a description of these data, the description was made jointly.

Some questions are still lacking when we discuss the treatment of pediatric mature B lymphomas, especially in the group of refractory/relapsed patients. Overall survival in this group of patients improves when consolidated with bone marrow transplant after immunochemotherapy treatment [Anoop et al. (38); Jourdain et al. (26)], especially when a full response is reached. Jourdain et al. (26) reported an OS of 54.3% in patients who underwent transplantation in these circumstances vs. 0%, *p* = 0.016. However, there are still questions about the best type of transplant that can be performed: autologous (the most commonly used type) or allogeneic? Furthermore, Anoop et al. (38) reported better OS in relapsed or refractory patients who received at least 4 doses of rituximab than in those who received two or more, similar to patients in first-line treatment: more trials are needed to evaluate whether this difference is confirmed. Finally, would it be beneficial if rituximab was used after bone marrow transplantation as a maintenance treatment?

Despite the ethical conflicts that this type of research can cause, the trial of Samochatova et al. (27), which showed good response in the treatment of patients undergoing first-line treatment with reduced doses of methotrexate when rituximab was used, may pave the way for other studies that have this purpose, with the intent of reducing late adverse effects, which are very harmful for survivors of pediatric neoplasms. As another example, we can cite the study of Goldman et al. (42), in which the intervention group demonstrated superiority with the inclusion of rituximab and reduction of the dose of anthracyclines in patients stratified as FAB Group B with stage I/II and stage III with lactic acid dehydrogenase <2X upper normal limits. Finally, regarding the use of rituximab in the Brazilian pediatric oncology and hematology institutions interviewed, we can observe that most of the institutions rely on their own financing or on the financing provided by nongovernmental organizations for the use of the medication with their patients. Notably, many patients have to file lawsuits to obtain this therapeutic agent. These alternatives to offer the best treatment for patients are praiseworthy, but we know that these financing alternatives may encounter setbacks that generate delays or failures in their administration and, consequently, jeopardize treatment. Our meta-analysis revealed a clear improvement in patient survival with the addition of rituximab to chemotherapy regimens in the population studied, and we believe that financing for the acquisition of rituximab starts being carried out in the same way as that for conventional chemotherapy drugs.

In conclusion, the results of our systematic review demonstrate that, in the vast majority of the included trials, either on first-line therapy or later, survival (event-free or overall) is greater when rituximab is employed in association with chemotherapy. Nevertheless, many of the trials had nonsignificant *p* values for these comparisons, probably due to the small sample sizes included.

Our meta-analysis revealed that there is a tangible benefit in the use of rituximab in the evaluation of events and deaths occurring in patients receiving first-line treatment (with the exception of PMBL). Furthermore, this benefit remains when evaluating the OS of patients with relapsed and refractory disease who use this medication.

## Data Availability

The original contributions presented in the study are included in the article/Supplementary Material, further inquiries can be directed to the corresponding author.
