# Supplementary material for: Use of rituximab in mature, high-grade and advanced-stage pediatric B-lineage non-Hodgkin lymphomas: a systematic review, meta-analysis and the Brazilian reality
Source: Front Pediatr. 2025 Jan 20;13:1532274. doi: 10.3389/fped.2025.1532274 (PMC11789686; doi:10.3389/fped.2025.1532274)
Supplement: Supplementary file 1 [file Table1.docx]

***Supplementary Material***

**SUPPLEMENTAL TABLE S1 Assessment of the included randomized study regarding risk of bias, according to RoB2 tool**

**
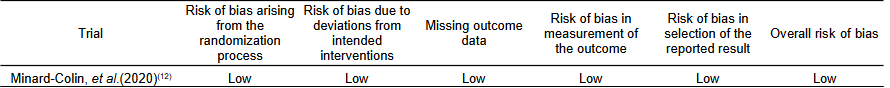
**

**SUPPLEMENTAL TABLE S2 Assessment of included non-randomized studies regarding risk of bias, according to ROBINS-I tool**


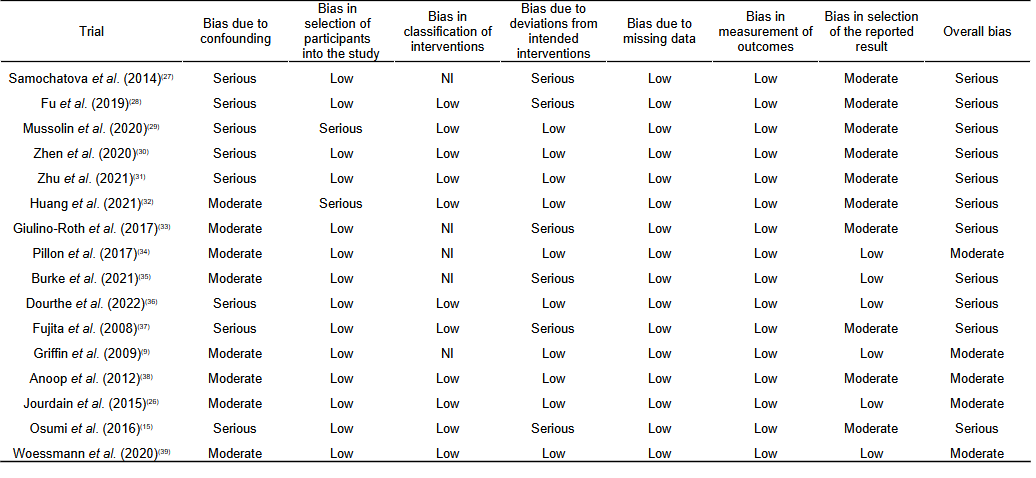
NI = No Information
